# Intimate partner violence management and referral practices of primary care workers in a selected population in Turkey

**DOI:** 10.1017/S1463423619000288

**Published:** 2019-06-25

**Authors:** Aysegul Catak Taskiran, Aysun Ozsahin, Tamer Edirne

**Affiliations:** Department of Family Medicine, Pamukkale University, Denizli, Turkey

**Keywords:** domestic violence, intimate partner violence, primary care, screening intimate partner violence

## Abstract

**Background:**

Violence against women is a significant public health problem and primary care workers (PCWs) have a crucial role in managing violence against women. However, though intimate partner violence (IPV) is frequently seen in primary care, most cases remain unreported.

**Aims:**

This study aims to investigate family physicians’ (FPs’) and co-working midwifes/nurses’ (M/Ns’) explanations about their responses to women disclosing IPV and the reasons for their actions.

**Methods:**

We conducted a cross-sectional survey via a face-to-face administered questionnaire interview involving 266 PCWs in a selected area in Turkey. We questioned the reasoning behind inappropriate responses such as not examining the patient and document findings, not recording a code of violence, and not notifying the police in the case of a disclosure of IPV.

**Results:**

We surveyed 129 FPs and 137 M/Ns. We found that the disclosure of IPV in primary care is very high, but more than one-third of physicians and half of M/Ns respond inappropriately. Reasons for inappropriate response varied. The majority believed that the victim would continue to live with her batterer, making any report ineffective. Some expressed concern for the women’s and their own personal safety, citing an increase in assault cases by perpetrators in the last few years. Many indicated a lack of knowledge about management of violence cases.

**Conclusion:**

Multiple barriers challenge PCWs in helping abused women. Common behaviours, safety concerns, and a lack of knowledge seem to be the major barriers to responding appropriately to IPV. To address this issue appropriately, protective measures for both parties – PCWs and violence victims – need to be enacted and a supportive constitutional and societal organization is required. Screening and identification should lead to interventions that benefit the victims rather than harming them.

## Introduction

Violence against women is an important community problem in both Turkey and abroad. Recent data from the World Health Organization revealed that 37% of women in the East Mediterranean region, including Turkey, are exposed to intimate partner violence (IPV) at some point during their lives (World Health Organization, 2014). This is supported by several local studies that have suggested there is a lifetime IPV rate of 29–44% among Turkish women (Sen and Bolsoy, 2017; HUIPS, 2015).

As compared with those in neighbouring countries, women in Turkey have far better legal protections against violence. The Law to Protect Family and Prevent Violence Against Woman and Children was established in Turkey on 8 March 2012 in agreement with the Council of Europe Convention on Preventing and Combating Violence against Women and Intimate Partner Violence (the Istanbul Protocol) (The Library of Congress, 2012). The aim of this law is to protect women, children, and vulnerable family members who have been subject to violence or who are at risk of experiencing violence, and to standardize procedures and principles with regard to the procedures of preventing the violence against those people.

Accordingly, governmental personnel are required to notify competent organizations or authorities if they identify women at risk of or exposed to IPV; otherwise, they face a prison sentence of up to one year according to the Turkish Criminal Code. According to a national report on the state of IPV against women in 2014 (Hacettepe, 2015), the most frequent admission sites of IPV victims are police stations followed by health institutions. The existing national practice guidelines for IPV intervention clearly describe the responsibilities of primary care workers (PCWs) in cases of suspected or verified violence. First, physicians are expected to record a ‘code of violence’ on the national electronic health record system, which notifies the local Health Directorate. This record also must contain the findings of the examination of the victim. Thereafter, a file of ‘intimate partner violence against women’ must be created to notify the local Directorate of Family and Social Policies and the Violence Prevention and Monitoring Centre (VPMC), which works on a 24/7 basis to support the victims. These centres are state-run shelters equipped with teams that include psychologists, psychological counsellors, social workers, lawyers, and nurses that provide housing; financial aid; and psychological, professional, legal, and social guidance. Women who are in need or who were referred by PCWs can obtain housing in these institutions with their children. At last, assessing the safety of the victim is recommended in order to discuss a safety plan including calling the police immediately if any danger indicators are present.

By the beginning of 2016, the number of VPMCs had increased to 40 in the country, and 60 522 women, 5126 men, and 84 100 children have benefited from their services. However, in addition to still being numerically insufficient, shelters seem to lag behind providing protective and supportive services in the way they are supposed to, mostly due to the unawareness or lack of knowledge of the employees (Tozlu and Goksel, 2016). In Turkey, access to the data recorded by governmental institutions on violence against women is limited. Furthermore, while criminal records are available, police records on IPV are not open to public access. The Ministry of Health (2014) shared 2013 health records indicating that 13 853 women applied to medical institutions complaining of injuries caused by IPV, but the number of official complaints remains unknown. In 2014, more than 13 000 cases of IPV against women were reported to gendarmerie forces (a branch of the police that operate in rural and semirural areas) that indicated the existence of more than 14 000 victims. Cases of violence against women and the number of victims are also recorded by other police forces; however, reliable statistics are not available (Dasre *et al.*, 2017).

PCWs in Turkey work at family health centres (FHCs), where at least one general practitioner (GP)/family physician (FP) and one assisting midwife/nurse (M/N) provide health care, with an average of four to five GPs/FPs present at the facility in total. These practices provide individually oriented primary care to the population on their lists. These PCWs are furthermore expected to assess violence victims, document findings, and provide medical care if necessary. However, despite the existence of a variety of legal arrangements in the Turkish legislation, the successful response to violence victims is dependent on providers’ knowledge, attitudes, and preparedness. Survey data from Turkish studies suggest that many health professionals lack the correct attitudes and knowledge to respond appropriately to women who have been subjected to violence (Duman *et al.*, 2016). In addition, the implementation of legal arrangements is in some way ineffective due to a lack of resources, monitoring systems, evaluation and follow-up measures, and inadequate support sites such as shelters (Tozlu and Goksel, 2016; CEDAW, 2014).

Although health policies encourage PCWs to address IPV and many precautions are established for the detection of and response to IPV victims, many women disclosing IPV still receive poor services worldwide (Colombini *et al.*, 2008) and PCWs often do not respond appropriately if IPV is disclosed (Taft *et al.*, 2004; Hegarty *et al.*, 2010). The most suggested reasons for this include a lack of IPV knowledge and training, a lack of time, and a lack of necessary resources (Ramsay *et al.*, 2012). Women subjected to IPV trust PCWs with their disclosures of violence (Feder *et al.*, 2006) and PCWs encounter women affected by violence frequently (Bonomi *et al.*, 2009).

The present study aimed to examine the explanations of FPs and M/Ns regarding their responses to women disclosing physical, verbal, or sexual violence in a selected population in Turkey and to explore the reasons behind their actions.

## Methods

We designed a cross-sectional survey with PCWs in a mid-sized town in Turkey, aiming to include all PCWs in the city centre. A list of all FHCs and PCWs located in the central district of the city was retrieved and all were visited and invited verbally by the authors. The Ethical Committee of Nonclinical Studies of the local university approved this study (24.05.2017/55517). A total of 30 FHCs with 316 PCWs consisting of 158 physicians and 158 M/Ns were approached and invited for the study, and 266 individuals ultimately participated (84.2% response rate) between June 2017 and December 2017. Thirty-six refused to participate (58.3% of them physicians, mostly due to heavy workload and/or no interest in taking part) and another 16 withdrew prior to data collection. All participants provided informed consent verbally. Recruited practices showed similar socioeconomic features between the study subjects and those who declined to participate.

### Instrument

A questionnaire was prepared according to existing data in the literature through searching using keywords relating to the topics of IPV, barriers to screening violence, primary care, so on. The resulting questionnaire started with items inquiring demographic characteristics like age, sex, work experience, and family status in addition to ever having witnessed an IPV case. In the second part, we asked them to remember the last case of a disclosure of physical, verbal, or sexual violence by a female patient and their response in this case. The act of the PCWs was accepted as appropriate when the response was ‘examined the patient and documented findings as violence’ for the physicians and ‘informed the physician’ for the M/Ns, respectively, followed by ‘notified the police’. All other responses were noted as inappropriate. The following part of the questionnaire attempted to elucidate the reasons for why they did not respond appropriately and pursue an IPV case. Participants were asked to choose only one item from the prepared answer list (Table 3). A pilot study with 10 PCWs was carried out to test the clarity, applicability, and feasibility of the questionnaire.

We approached GP/FPs and M/Ns privately and explained to them that all data collected will be used anonymously. We chose a face-to-face method through writing on paper to give explanations when requested. Surveys were completed in a private room in the FHC and took approximately 10–12 min.

### Statistical analysis

Descriptive statistics and Pearson chi-squared tests were used to associate demographic characteristics of physicians and M/Ns. nonparametric Mann–Whitney U tests were used to compare physicians’ and M/Ns’ responses.

Data were entered into the Statistical Package for the Social Sciences for Windows version 16.0 software program (IBM Corp., Armonk, NY, USA) for the calculation of frequencies as percentages and averages as means or medians and standard deviations (SDs) or interquartile ranges (IQRs).

## Results

Two hundred sixty-six primary health care workers completed the survey. Their mean age was 42.6 (SD: 6.52) years (range: 29–56 years) and 65.8% (175) were female. Most of them were married and the majority had one to two children on average (Table 1).

**Table 1.** Demographic characteristics of recruited primary health care workers, Turkey 2017

	FPs (*n* = 129)	M/Ns (*n* = 137)
Female sex (%)	33.1	97.1
Age, mean years (SD)	46 (6.34)	39 (5.98)
Mean working years (SD)	19.8 (6.89)	14.2 (6.57)
Having children, *n* (%)		
0	11 (8.6)	9 (6.8)
1–2	106 (83.5)	105 (79.5)
≥3	10 (7.9)	18 (13.7)
Married, *n* (%)	115 (89,1)	124 (90.5)
Ever witnessed IPV, *n* (%)	124 (96.1)	114 (83.2)

In the previous year, 89.5% (238/266) of health workers had encountered one or more cases of IPV. In addition, 63.6% of FPs and 41.6% of M/Ns reported to have experienced one to three cases of IPV disclosure at their workplace, while three FPs reported more than 10 cases (Table 2).

**Table 2.** Disclosure rates of IPV to recruited PCWs at work in the previous year, Turkey 2017

	1–3	4–6	7–9	≥10	Never
FPs *n* (%)	82 (63.8)	14 (10.9)	1 (0.8)	3 (2.3)	29 (22.5)
M/Ns *n* (%)	57 (41.6)	6 (4.4)	1 (0.7)	–	73 (53.3)

Overall, 52.6% of all PCWs responded appropriately to a disclosure. The proportion of FPs who examined the patient, documented the findings, and informed the police was 64.3%, while 41.6% of the M/Ns informed the physician or the police. In the remaining cases, the action most commonly taken was advising the patient to apply to a judicial institution or to a VPSC; however, a small group ignored the case.

When asked for their reasons behind not reporting IPV, the majority believed that the abused women would return to her batterer; therefore, it would make no sense to file a report. A lack of knowledge in detecting, documenting, and referring IPV cases also played an important role in their responses. Fourteen percent of responders did express concerns for their own personal safety, and 12% complained of the absence of security personnel at the workplace (Table 3).

**Table 3.** Reasons stated by recruited PCWs not to document and report IPV cases encountered, Turkey 2017

	*n*	%
Believing the abused women would not leave her partner	96	36.0
Lack of knowledge in detecting violence findings	48	18.0
Concerns for the women’s and their own safety	39	14.6
Absence of guards and police officers at the FHCs	34	12.8
Lack of knowledge in preparing a document of violence	19	7.1
Lack of knowledge in referral process	18	6.8
Didn’t thought it was important	6	2.2
Victim may have ‘done something’ to bring about the violence	6	2.2
Total	266	100

## Discussion

We found that the disclosure of IPV to our population of PCWs was very high. However, our cohort seems to fail to respond appropriately with regard to the law in many cases. When encountering violence, just more than half of all PCWs responded appropriately by recording a ‘code of violence’, which initiates a referral of the victim to competent organizations or authorities.

We observed that 96% of GPs/FPs and 83% of M/Ns in our study have encountered at least one IPV case ever in their career to date. In the previous 12 months, 64% of our GP/FPs were confronted with a victim of IPV between one and three times. Researchers from the United Kingdom have reported that 71% of primary care physicians had diagnosed one or more cases of IPV in the last month in 2012 (Ramsay *et al.*, 2012), while 37% of Scottish GPs stated they had encountered at least one IPV case in 2010 (Cairns *et al.*, 2005).

Prevalence rates of IPV in health care settings were reported by women who attended health care centres in Syria and Jordan as 15% and 87%, respectively, and the majority testified that the abuse was from their husbands (Maziak *et al.*, 2003; Al-Nsour *et al.*, 2009).

Although PCWs are believed to be ideally situated to screen and respond to the disclosure of violence and abused women largely want to be screened for IPV by their primary care physicians (Black, 2011), it appears that many IPV cases are not identified and the appropriate management of abused women is low in primary care settings worldwide (Morse *et al.*, 2012).

Despite the importance of the management of IPV in primary care settings, there are several barriers to delivering optimum care for IPV victims. In this study, we found that FPs recorded a ‘code of violence’ in only 64% of the IPV cases they encountered, while M/Ns informed the physician in only 41% of the IPV cases they acknowledged. A study from the United States in 2014 reported that, of physicians who screened patients for IPV, the vast majority (81.5%) did not act appropriately, such as offering a follow-up appointment or making a referral to IPV facilities (Sutherland *et al.*, 2014). In a study reviewing affirmed IPV cases, researchers found that IPV was reported by health institutes in only 31 (9.1%) cases of the 337 records reviewed (Magnussen *et al.*, 2004). There is accumulating evidence in the literature that, when women do disclose violence, their health care providers often do not give them the necessary support and information (McCall-Hosenfeld *et al.*, 2014; Garcia-Moreno *et al.*, 2015).

We identified that there are barriers at multiple levels to appropriate management. Many physician-level barriers to screen women for violence have been described already in primary care, including inadequate training in the care of IPV cases, a lack of physician confidence in addressing IPV, and a lack of specialized support systems to assist PCWs in managing victims identified by screening (Gerber *et al.*, 2005; Jaffee *et al.*, 2005; Colarossi *et al.*, 2010). Researchers have examined the reasoning behind low rates of screening previously and quoted barriers including lack of time (Colarossi *et al.*, 2010), lack of knowledge regarding public resources, feelings of ineffectiveness (Elliott *et al.*, 2002; Colarossi *et al.*, 2010), underestimation of the consequences of IPV, and negative results in the relationship with patients (Elliott *et al.*, 2002).

Although the reasons for inappropriate management in IPV cases were reported to be similar, this study was designed to explore the actions of primary PCWs in the case of a disclosure, not screening practices. In this study, the majority believed that the abused women would return to their batterers and, therefore, it did not make any sense to record or report the presence of IPV. There are several aspects that may clarify this belief in Turkey.

Traditional opinions concerning family privacy, family unity, and gender roles were found to have posed problems to PCWs in the management of IPV cases. In patriarchal societies formed by religious doctrines and directed by male authorities, existing attitudes and systems endorse and reinforce male dominance, resulting in beliefs that portray women as naturally inferior or obedient to men (Sakalli-Ugurlu and Akbas; 2013; Carter, 2014). This patriarchal and traditionalist structure results in women’s dependency on men, owing to their home care tasks, lack of an income, lack of education and job skills, and lack of health insurance. Thus, women often are not given the option to consent or dissent, nor do they really have much control over their lives overall. Such a dependency is itself a main causal motive in the perpetuation of male violence against women (Gul, 2013). Women with more financial resources and education may have greater independence in conceptualizing certain situations as unacceptable or intolerable and seeking external support (Goodman *et al.*, 2009). We were not able to inquire about the status of the women PCWs encountered, but data provide information that most of the women in Turkey are working for the family benefit and are caretakers of their children, husband, and elderly or ill family members (Dedeoglu and Ozturk, 2010).

In a large study in Turkey in 2013, it was stated that husbands have higher educational levels than their wives do, and the main reasons for women not working are their status as a housewife and caretaker of children or them not being allowed to work. In the same study, 13% of the women agreed that physical violence is justified in case a woman neglects the children or argues with her husband (HUIPS, 2014a).

Noting that women in Turkey are less educated and more economically dependent upon their husbands and more likely to normalize IPV (Efe and Ayaz, 2010), we surmise that they are also less able to respond effectively to IPV and may express ambivalence about leaving, which might explain the acts of our PCWs (Morse *et al.*, 2012). They may consider the violence to be a common behaviour and are afraid that interference will only cause more problems for the battered women, who have no choice other than returning to their families and husbands (Duman *et al.*, 2016; Tekkas and Betrus, 2018).

In Turkey, an inefficient *justice system response* is another significant systemic barrier to the reporting of IPV cases, which has also been documented in other research (Alobaty *et al.*, 2013). Police reluctance to arrest IPV committers without sufficiently evidenced physical injury, indifference, lack of empathy, and sexist approaches from justice personnel along with long and exhausting administrative stages in the legal service system led to dissatisfaction among affected women (Mor Cati, 2010; HUIPS, 2014b). Negative police and justice system responding (eg, attempts to reconcile, inadequate prosecution policies) and restraining orders or court interventions that do not help (or make things worse) results in many victims continuing to suffer silently. Moreover, despite a broad range of legal, medical, and public initiatives aiming to stop IPV, associated killings are increasing in Turkey, with accumulating cases of recurrent violence directed against the battered women after they press charges against their partners. Despite the new regulations and supportive resources available, partners or former partners killed 1703 women in 2011 in Turkey (Aydin *et al.*, 2016), and the real numbers are estimated to be higher. This may partly reflect women’s unwillingness to report abuse, but some may suggest that this also reflects their lack of faith in the authorities responding appropriately to their needs as victims of crime. Indeed, the continuing prevalence of IPV has led to a generalized understanding that IPV victims’ needs are not adequately met by health and legal services (Boyacioglu, 2016).

A lack of critical safety measures including a negative *response of the family* and *the justice system* and lack of *responsiveness of community resources* for the victim before confronting a batterer may justify this high rate of inappropriate management in our study.

Personal safety concerns were the second most important reason for the participants not addressing IPV in our study. PCWs are concerned for their own safety and complain of the absence of security staff members at the workplace in general in Turkey (Usta *et al.*, 2014). Sufficient security at the workplace is vital in Turkey, where assaults against PCWs, especially physicians, are growing (Smith, 2015). It is understandable that PCWs have concerns for their own safety due to the extensive onslaught of violence against doctors in the world and there existing a lack of protective measures (Magnavita and Heponiemi, 2012; Algwaiz and Algahim, 2012). Although some results from high-income countries mention that well-trained PCWs can address IPV effectively and improve outcomes (Wong *et al.*, 2006; Hegarty *et al.*, 2008), PCWs need to know that there are appropriate and supportive public resources and responsive police and legal institutions to manage IPV before reporting.

In this study, lack of knowledge in managing an IPV case was stated as the third most important reason for not reporting IPV. Inexperience in detecting violence findings and preparing a medical record were also limitations stated by the PCWs. The need for education for PCWs in dealing with barriers to screening has been recognized in the literature frequently, citing unsatisfactory training and experience (Waalen *et al.*, 2000; Black, 2011).

Some women may choose to disclose to a midwife or nurse about their abuse, requiring that all health care members are prepared for addressing IPV appropriately. We found that disclosures to the M/Ns in our study were lower in number than disclosures to our physicians and that the M/Ns did not report more than the half of the cases they encountered. Researchers have shown that most of the nurses working in primary health care are inadequately equipped to detect and manage violence against women (Sundborg *et al.*, 2012).

There are some limitations to this study. First, it was restricted to the primary care setting and, thus, the results may not be generalizable to other sites. Second, its cross-sectional design based on data gathered via memory recall limits the reliability of our conclusions. Furthermore, although a pilot study was performed, our questionnaire was not further validated or standardized. Additional studies with a focus on the attitudes of PCWs regarding their actions when encountering IPV cases are necessary.

## Conclusion

If protective legislations or other public resources are not available, PCWs may ignore the subject of IPV. The absence of a system of care that facilitates and supports screening and reporting IPV may add a significant risk to women’s safety in resource-poor settings and in societies characterized by higher levels of gender inequality that resemble Turkey. In addition, there are little data to decide whether mandatory reporting of IPV cases increases or decreases safety. For routine IPV inquiry to be accepted as standard medical practice, it is important to demonstrate that the health care response benefits the victims while avoiding harm. It may be necessary to identify and study other outcomes, including safety, with a particular focus on low-income and middle-income settings.

## References

[ref1] Algwaiz WM and Algahim SA (2012) Violence exposure among healthcare professionals in Saudi public hospitals. a preliminary investigation. Saudi Medical Journal 33, 76–82. 22273653

[ref2] Al-Nsour M , Khawaja M and Al-Kayyali G (2009) İntimate partner violence against women in Jordan: evidence from health clinics. Journal of Family Violence 24, 569–575.

[ref3] Alobaty IY , Alkandari BA , Alshamali KA , Kamel MI and El-Shazly MK (2013) Barriers for intimate partner violence screening in primary health care centers. Alexandria Journal of Medicine 49, 175–180.

[ref5] Aydın BN , Kocagazioglu SY , Arda OE and Golge ZB (2016) Comparison of news about violence towards women in Media. Türk Psikoloji Yazıları 19, 64–75(Special Issue).

[ref6] Black MC (2011) Intimate partner violence and adverse health consequences. American Journal of Lifestyle Medicine 5, 428–439.

[ref7] Bonomi AE , Anderson ML , Rivara FP and Thompson RS (2009) Health care utilization and costs associated with physical and nonphysical-only intimate partner violence. Health Services Research 44, 1052–1067. 19674432 10.1111/j.1475-6773.2009.00955.x PMC2699921

[ref8] Boyacioglu I (2016) Violence against women and the past and present of women’s studies in Turkey: a call for psychological research. Türk Psikoloji Yazıları 19, 126–145 (Special Issue).

[ref9] Cairns AM , Mok JYQ and Welbury RR (2005) The dental practitioner and child protection in Scotland. British Dental Journal 8, 517–52077. 10.1038/sj.bdj.4812809 16244627

[ref11] Carter J (2014) A call to action: religion, women, violence and power. New York: Simon and Schuster.

[ref12] Colarossi L , Breitbart V and Betancourt G (2010) Barriers to screening for intimate partner violence: a mixed-methods study of providers in family planning clinics. Perspectives on Sexual and Reproductive Health 42, 236–243. 21126299 10.1363/4223610

[ref13] Colombini M , Mayhew S and Watts C (2008) Health-sector responses to intimate partner violence in low- and middle-income settings: a review of current models, challenges and opportunities. Bull World Health Organ 86, 635–642. 18797623 10.2471/BLT.07.045906 PMC2649453

[ref14] Dasre A , Greulich A and Ceren I (2017) Combating intimate partner violence against women in Turkey. The role of women’s economic empowerment. Documents de travail du Centre d’Economie de la Sorbonne.

[ref15] Dedeoglu S and Ozturk MY (2010) Endüstriyel Üretimde Kadın ve Göçmen Emeği: Ataerkillik ve Enformel Emek (in Turkish). Istanbul: Sosyal Arastırmalar Vakfı.

[ref16] Duman NB , Buyukgonenc L , Gungor T , Yilmazel G , Topuz S and Kocak DY (2016) Perception of violence against women among health care professionals and affecting factors. Jinekoloji - Obstetrik ve Neonatoloji Tıp Dergisi 13, 154–159.

[ref17] Efe SY and Ayaz S (2010) İntimate partner violence against women and women’s opinions related to intimate partner violence. Anatolian Journal of Psychiatry 11, 23–29.

[ref18] Elliott L , Nerney M , Jones T and Friedmann PD (2002) Barriers to screening for intimate partner violence. Journal of General Internal Medicine 17, 112–116. 11841526 10.1046/j.1525-1497.2002.10233.x PMC1495014

[ref19] Feder GS , Hutson M , Ramsay J and Taket AR (2006) Women exposed to intimate partner violence: expectations and experiences when they encounter health care professionals: a meta-analysis of qualitative studies. JAMA Internal Medicine 166, 22–37. 10.1001/archinte.166.1.22 16401807

[ref21] García-Moreno C , Hegarty K , d’Oliveira A , Koziol-McLain J , Colombini M and Feder G (2015) Violence against women and girls 2. The health-systems response to violence against women. Lancet 385, 1567–1579. 25467583 10.1016/S0140-6736(14)61837-7

[ref22] Gerber MR , Leiter KS , Hermann RC and Bor DH (2005) How and why community hospital clinicians document a positive screen for intimate partner violence: a cross-sectional study. BMC Family Practice 19, 48. 10.1186/1471-2296-6-48 PMC1318461 16297245

[ref24] Goodman LA , Smyth KF , Borges AM and Singer R (2009) When crises collide: how intimate partner violence and poverty intersect to shape women’s mental health and coping? Trauma Violence Abuse 10, 306–329. 19776085 10.1177/1524838009339754

[ref25] Gul SS (2013) The role of state in protecting women against intimate partner violence and women’s shelters in Turkey. Women’s Studies International Forum 38, 107–116.

[ref26] Hacettepe University Institute of Population Studies (Turkey) (HUIPS) (2014a) 2013 Turkey Demographic and Health Survey. Ankara, Turkey: Hacettepe University Institute of Population Studies, T.R. Ministry of Development and TÜBİTAK.

[ref27] Hacettepe University Institute of Population Studies (Turkey) (HUIPS) (2014b) Hacettepe University Institute of Population Studies. Research on violence against women in Turkey, 2014 Report. Retrieved 14 August 2018 from http://www.hips.hacettepe.edu.tr/KKSA-TRAnaRaporKitap26Mart.pdf

[ref28] Hacettepe University Institute of Population Studies (Turkey) (HUIPS) (2015) Hacettepe University Institute of Population Studies. Research on intimate partner violence against women in Turkey. Retrieved 28 March 2018 from http://www.hips.hacettepe.edu.tr/ING_SUMMARY_REPORT_VAW_2014.pdf

[ref30] Hegarty KL , Gunn JM , O’Doherty LJ , Taft A , Chondros P , Feder G , Astbury J and Brown S (2010) Women’s evaluation of abuse and violence care in general practice: a cluster randomised controlled trial (weave). BMC Public Health 10, 2. 20044929 10.1186/1471-2458-10-2 PMC2823699

[ref31] Hegarty KL , O’Doherty LJ , Gunn J , Pierce D and Taft A (2008) A brief counseling intervention by health professionals utilising the ‘readiness to change’ concept for women experiencing intimate partner abuse: The weave project. Journal of Family Studies 14, 376–388.

[ref32] Jaffee KD , Epling JW , Grant W , Ghandour RM and Callendar E (2005) Physician-identified barriers to intimate partner violence screening. Journal of Women’s Health 14, 713–720. 10.1089/jwh.2005.14.713 16232103

[ref35] Magnavita N and Heponiemi T (2012) Violence towards healthcare workers in a public health care facility in Italy: a repeated cross-sectional study. BMC Health Services Research 1291, 108. 10.1186/1472-6963-12-108 PMC3464150 22551645

[ref36] Magnussen L , Shoultz J , Oneha MF , Hla MM , Brees-Saunders Z , Akamine M , Talisayan B and Wong E (2004) Intimate-partner violence: a retrospective review of records in primary care settings. Journal of the American Association of Nurse Practitioners 16, 502–512. 10.1111/j.1745-7599.2004.tb00430.x 15617364

[ref37] Maziak W and Asfar T (2003) Physical abuse in low-income women in Aleppo, Syria. Health Care for Women International 24, 313–326. 12746003 10.1080/07399330390191689

[ref38] McCall-Hosenfeld JS , Weisman CS , Perry AN , Hillemeier MM and Chuang CH (2014) “I just keep my antennae out”: how rural primary care physicians respond to intimate partner violence. Journal of Interpersonal Violence 29, 2670–2694. 24424251 10.1177/0886260513517299 PMC4121375

[ref40] Ministry of Health (2014) Health Statistics Yearbook. Retrieved 13 February 2019 from https://sbu.saglik.gov.tr/Ekutuphane/kitaplar/EN%20YILLIK.pdf

[ref41] Mor Cati Women’s Shelter Foundation (Mor Çatı Kadın Sığınağı Vakfı) (2010) April 2010-December 2011 Report. Retrieved 14 August 2018 from https://www.morcati.org.tr/images/files/Nisan2010Aralik2011izlemeraporu.pdf

[ref42] Morse D , Ross L , Fogarty C , Mittal M and Cerulli C (2012) “They told me to leave”: how health care providers address intimate partner violence. Journal of the American Board of Family Medicine 12, 333–342. 10.3122/jabfm.2012.03.110193 PMC3388120 22570397

[ref45] Ramsay J , Rutterford C , Gregory A , Dunne D , Eldridge S , Sharp D and Feder G (2012) Intimate partner violence: knowledge, attitudes, and clinical practice of selected UK primary care healthcare clinicians. British Journal of General Practice 62, e647–e655. 10.3399/bjgp12X654623 PMC3426604 22947586

[ref46] Sakallı-Uğurlu N and Akbas G (2013) Namus kültürlerinde “Namus” ve Namus adına kadına siddet”: Sosyal psikolojik açıklamalar. Türk Psikoloji Yazıları 16, 76–91.

[ref47] Sen S and Bolsoy N (2017) Violence against women: prevalence and risk factors in Turkish sample. BMC Women’s Health 17, 100. 29100515 10.1186/s12905-017-0454-3 PMC5670523

[ref48] Smith M (2015) Rise in violence against doctors in Turkey, elsewhere. CMAJ 187, 643. 25991838 10.1503/cmaj.109-5062 PMC4467925

[ref49] Sundborg EM , Saleh-Stain N , Wandell P and Tornkvist L (2012) Nurse’s preparedness to care for women exposed to intimate partner violence: a quantitative study in primary health care. BMC Nursing 11, 1–11. 22233776 10.1186/1472-6955-11-1 PMC3293728

[ref50] Sutherland MA , Fontenot HB and Fantasia HC (2014) Beyond assessment: examining providers’ responses to disclosures of violence. Journal of the American Association of Nurse Practitioners 26, 567–573. 24420743 10.1002/2327-6924.12101

[ref51] Taft A , Broom D and Legge D (2004) General practitioner management of intimate partner abuse and the whole family: a qualitative study. BMJ 328, 618–621. 14766719 10.1136/bmj.38014.627535.0B PMC381135

[ref52] Tekkas KK and Betrus P (2018) Violence against women in turkey: a social ecological framework of determinants and prevention strategies. Trauma Violence Abuse 1, 1524838018781104. 10.1177/1524838018781104 29888680

[ref53] The Convention on the Elimination of all Forms of Discrimination Against Women (CEDAW) (2014) The convention on the elimination of all forms of discrimination against women 7. Periodic Country Report. Retrieved 30 November 2018 from https://kadininstatusu.aile.gov.tr/uploads/pages/ulusarasi-belgeler-kuruluslar/cedaw-7-ulke-raporu-list-of-issues-yanitlar-ingilizce.pdf.

[ref54] The Library of Congress (2012) Global Legal Monitor. Wendy Zeldin. Turkey: Parliament Adopts Law on Prevention of İntimate partner Violence. Retrieved 29 September 2018 from https://www.loc.gov/law/foreign-news/article/Turkey-parliament-adopts-law-on-prevention-of-intimatepartner-violence/

[ref55] Tozlu C and Goksel A (2016) WAVE: Violence against Women Country Report Turkey.

[ref56] Usta J , Feder G and Antoun J (2014) Attitudes towards intimate partner violence in Lebanon: a qualitative study of primary care practitioners. British Journal of General Practice 64, e313–e320. 10.3399/bjgp14X680077 PMC4032013 24868068

[ref57] Waalen J , Goodwin MM , Spitz AM , Petersen R and Saltzman LE (2000) Screening for intimate partner violence by health care providers: barriers and interventions. American Journal of Preventive Medicine 19, 230–237. 11064226 10.1016/s0749-3797(00)00229-4

[ref60] Wong SLF , Wester F , Mol SS and Lagro-Janssen TL (2006) Increased awareness of intimate partner abuse after training: a randomized controlled trial. British Journal of General Practice 56, 249–257. PMC1832231 16611512

[ref61] World Health Organization (WHO) (2014) Global status report on violence prevention. WHO library cataloguing-in-publication data. Retrieved 30 March 2018 from http://www.who.int/violence_injury_prevention/violence/status_report/2014/en/

